# Correction: *Zika Virus*-Derived E-DIII Protein Displayed on Immunologically Optimized VLPs Induces Neutralizing Antibodies without Causing Enhancement of *Dengue Virus* Infection. *Vaccines* 2019, *7*, 72

**DOI:** 10.3390/vaccines8010094

**Published:** 2020-02-20

**Authors:** Gustavo Cabral-Miranda, Stephanie M. Lim, Mona O. Mohsen, Ilya V. Pobelov, Elisa S. Roesti, Matthew D. Heath, Murray A. Skinner, Matthias F. Kramer, Byron E. E. Martina, Martin F. Bachmann

**Affiliations:** 1 The Jenner Institute, Nuffield Department of Medicine, Centre for Cellular and Molecular Physiology (CCMP), University of Oxford, Oxford OX1 2JD, UK; mmohsen@qf.org.qa; 2 Immunology, RIA, Inselspital, University of Bern, 3010 Bern, Switzerland; elisa.roesti@dbmr.unibe.ch; 3 Artemis Bio-Support, Molengraaffsingel, 2629 Delft, The Netherlands; s.lim@artemisonehealth.com (S.M.L.); b.martina@artemisonehealth.com (B.E.E.M.); 4 Department of Chemistry and Biochemistry, University of Bern, 3010 Bern, Switzerland; ilya.pobelov@dcb.unibe.ch; 5 Bencard Adjuvant Systems, Worthing BN14 8SA, UK; matthew.heath@allergytherapeutics.com (M.D.H.); Murray.Skinner@allergytherapeutics.com (M.A.S.); KramerM@bencard.com (M.F.K.); 6 Department of Viroscience, Erasmus Medical Center, 3015 GD Rotterdam, The Netherlands

The authors wish to make the following correction to their paper [1]. 

The same image was mistakenly selected for Figure 2 and Figure 3. The image became replaced as you see in Figure 3 below. 

## Figures and Tables

**Figure 2:**
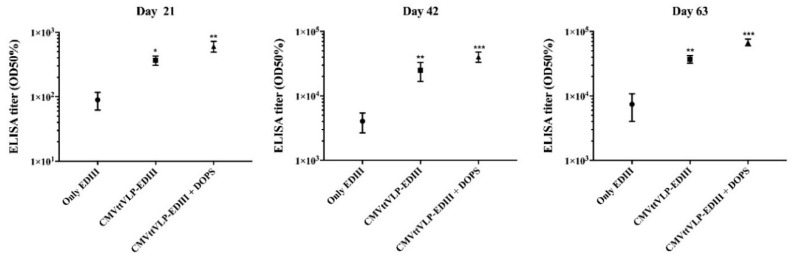
CuMVtt-EDIII formulated in DOPS indices highest total IgG responses. The figure shows IgGs level of three different groups of mice vaccinated with EDIII alone formulated in phosphate-buffered saline (PBS), with vaccine formulated by coupling EDIII to VLP (CuMVtt-EDIII) or formulating CuMVtt-EDIII plus DOPS adjuvant. The assessment was done with sera collected three weeks after each vaccination. The results were analyzed using GraphPad Prism software applied to assess the means of three groups by one-way analysis of variance (ANOVA). The values observed in the negative control group (vaccinated with only PBS) were subtracted from the titers of the other experimental groups. Note: * *p* < 0.01, ** *p* < 0.001, *** *p* < 0.0001.

**Figure 3:**
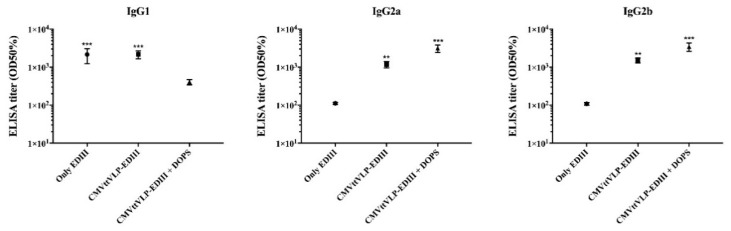
Subclasses of IgG. The figure shows the specific subclasses of IgG induced by each vaccine from samples collected three weeks after the third vaccination (Day 63). The group of mice vaccinated with only EDIII predominantly produced IgG1 while EDIII coupled to VLP (CuMVtt-EDIII) also induced IgG2a and IgG2b. When DOPS adjuvant was ad-mixed, this skewing towards IgG2a and b was even more pronounced. The results were analyzed using GraphPad Prism software applied to assess the means of three groups by one-way analysis of variance (ANOVA). The values observed in the negative control group were subtracted from the titers of the other experimental groups. Note: ** *p* < 0.001, *** *p* < 0.0001.

## References

[1] Cabral-Miranda G., Lim S.M., Mohsen M.O., Pobelov I.V., Roesti E.S., Heath M.D., Skinner M.A., Kramer M.F., Martina B.E.E., Bachmann M.F. (2019). *Zika Virus*-Derived E-DIII Protein Displayed on Immunologically Optimized VLPs Induces Neutralizing Antibodies without Causing Enhancement of *Dengue Virus* Infection. Vaccines.

